# Preparation of Multilayered Polymeric Structures Using a Novel Four-Needle Coaxial Electrohydrodynamic Device

**DOI:** 10.1002/marc.201300777

**Published:** 2014-02-08

**Authors:** Sheyda Labbaf, Hanif Ghanbar, Eleanor Stride, Mohan* Edirisinghe

**Affiliations:** Department of Mechanical Engineering, Biomaterials Research Lab, University College LondonTorrington Place, London, WC1 7JE, UK; Department of Mechanical Engineering, University College LondonTorrington Place, London, WC1E 7JE, UK; Institute of Biomedical Engineering, Oxford UniversityOxford, OX3 7DQ, UK

**Keywords:** coaxial device, electrohydrodynamic, multilayered structures, polymers

## Abstract

Coaxial four-needle electrohydrodynamic forming is applied for the first time to prepare layered structures in both particle and fiber form. Four different biocompatible polymers, polyethylene glycol, poly (lactic-*co*-glycolic acid), polycaprolactone, and polymethylsilsesquioxane, are used to generate four distinct layers confirmed using transmission and scanning electron microscopy combined with focused ion beam milling. The incorporation and release of different dyes within the polymeric system of four layers are demonstrated, something that is much desired in modern applications such as the polypill where multiple active pharmaceutical ingredients can be combined to treat numerous diseases.

## 1. Introduction

Electrohydrodynamic (EHD) forming has shown considerable promise for the production of fibers and particles at the micro- and nanoscale. Although the underlying physics has been well known for many decades, EHD engineering and technology has only become established in recent years. Due to the increased demand for multilayered structures, there is still need for further changes or enhancement to the current state-of-art technique to make it more suitable for desired applications. EHD enables the production of complex structures in both nano and micrometer size range.^1^ The main advantages of EHD processing over other conventional encapsulation methods are the avoidance of the use of excessive amounts of surfactants, high temperatures, pressures, or shear rates.^2^,^3^ The apparatus is relatively low cost and simple to operate and particles with a narrow size distribution can be generated in a single step with high encapsulation integrity. By utilizing a coaxial system, whereby two or more materials are processed simultaneously, multilayered structures can be produced. Such structures have a wide range of applications; in drug delivery for example, layering can be used to enable release of therapeutic agents at variable rates.^4^–^6^

Nevertheless EHD coaxial systems have been limited to two needles for decades until Edirisinghe et al. demonstrated the use of a three-needle coaxial device, first as a proof of concept study and more recently to prepare actual tri-layered spherical polymeric particles.^3^ However, going further to generate more layers by EHD in different morphologies will be a significant leap as it is greatly desired in biomedical applications such as drug delivery and tissue engineering.^4^,^5^,^7^–^11^ The current study demonstrates, for the first time, the feasibility of using a four-needle coaxial device to prepare four-layered structures in both particle and fiber form. These were prepared in a single step under ambient conditions, which are hugely beneficial in preventing the degradation of active pharmaceutical ingredients. For this purpose, in this study, four biocompatible polymers, namely polyethylene glycol (PEG), poly (lactic-*co*-glycolic acid) (PLGA), poly­caprolactone (PCL), and polymethylsilsesquioxane (PMSQ), were used as all have received significant attention for a range of medical applications.^12^–^14^

## 2. Experimental Section

### 2.1 Materials

PMSQ powder (molecular weight 7465 g mol^−1^) was provided by Wacker Chemie AG, GmbH, Burghausen, Germany. PLGA-co-polymer 50:50 Resomer RG503H, molecular weight 33 000 g mol^−1^ was purchased from Boehringer Ingelheim, Germany. Evans blue, pyronin B, pinacyanol chloride, hematoxylin, PCL (molecular weight 45 000 g mol^−1^), PEG (molecular weight 18 000 g mol^−1^), and solvents including ethanol (EtOH), dimethyl carbonate (DMC), dichloromethane (DCM) were all purchased from Sigma–Aldrich (Poole, UK).

Polymer solutions were prepared by mixing polymer with appropriate solvent, based on Table1 (weight ratios), and stirring for 900 s at ambient conditions. In order to measure solution properties including viscosity, surface tension, and electrical conductivity, the following calibrated procedures were applied: VISCOEASY rotational viscometer (Brookfield Rheometer) was used to measure viscosity. Surface tension was measured using a Kruss Tensiometer (Standard Wilhelmy's plate method). Electrical conductivity was measured using a HI-8733 (Hanna Instruments, USA) conductivity probe. All solution properties are listed in Table1.

**Table 1:** Physical properties of the solutions and solvents used in the current study followed by standard deviation values (±)

Sample	Viscosity [mPa s]	Surface tension [mN m^−1^^]^	Electrical conductivity x 10^−4^ [S m^−1^]
Particle			
DCM:PEG 90:10	3.3 ± 0.01	25.3 ± 0.6	92 ± 1.5
DMC:PLGA 95:5	3.1 ± 0.1	27.7 ± 1.8	0.2 ± 0
DCM:PCL 97:3	6.3 ± 0.2	24.7 ± 0.5	0.01 ± 0
EtOH:PMSQ 88:12	1.6 ± 0.1	22.5 ± 0.4	9 ± 0.6
Fiber			
DCM:PEG 75:25	12.4 ± 0.01	26.9 ± 0.6	89 ± 0.4
DMC:PLGA 90:10	6.7 ± 0.2	29 ± 0.5	0.2 ± 0
DCM:PCL 92:8	12.3 ± 0.7	27.5 ± 0.4	0.01 ± 0
EtOH:PMSQ 75:25	4.4 ± 0.2	24.4 ± 1.0	13 ± 0.3

### 2.2 Forming Process

EHD processing, which in the context of materials forming allows the generation of products (relics) from droplets, requires the application of a potential difference (applied voltage) to a flowing medium to transfer electric charge to the liquid to form a jet.^2^ Generally, a minimum solution electrical conductivity of ≈0.01 μS m^−1^ is desired for EHD processing^15^ and we satisfy this in the selection of solvents and polymers (Table1). Nevertheless, surface tension and viscosity of the working solutions are the main parameters in controlling the window at which a particular polymer/solvent combination can be EHD processed.^2^

**Figure 1:**
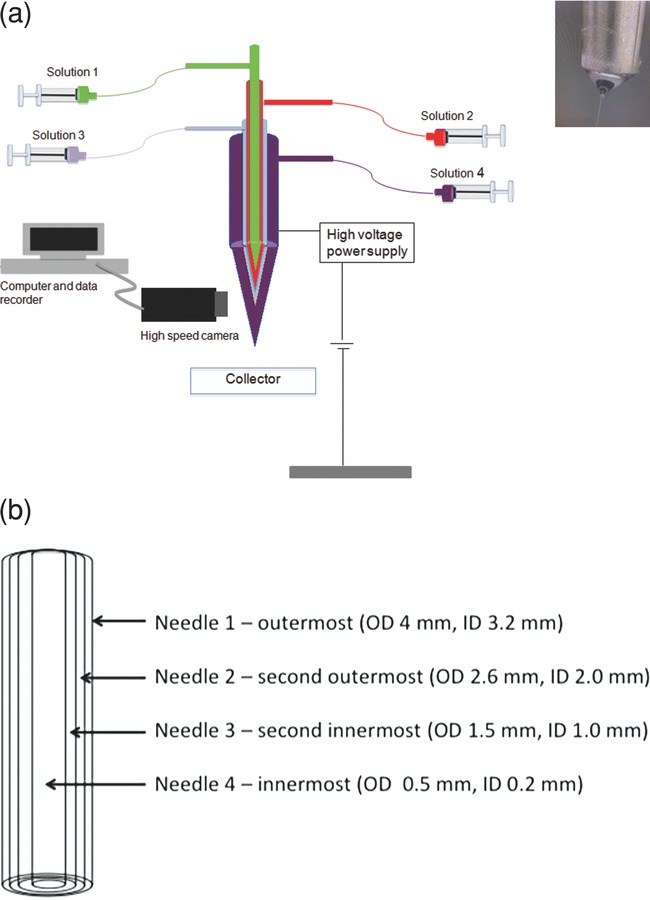
Schematic illustration of a) the experimental set-up of the EHD process using a four-needle device for forming of four-layer structures with a stable jet (inset) and b) the coaxial needle arrangement with labeled dimensions, where ID and OD are internal and outer diameters, respectively.

The experimental set-up for this study is shown in Figure 1. The processing parameters were varied to enable, separately, fiber, and particle formation. Four coaxial liquid streams were produced simultaneously to form multilayered fibers and particles and the conditions applied for the preparation of these are summarized in Table2. For the preparation of fibers, more concentrated polymer solutions were required, and as a consequence, a higher voltage is required to overcome higher viscosity (Table1) as the electrostatic force draws the solution from the needles.

**Table 2:** Summary of the processing conditions used. The flow rates quoted are for needles 1,2,3, and 4, respectively. Working distance is from needle tip to collector

Structure	Voltage [kV]	Flow rate [μL min^−1^]	Working distance [mm]
Particle	9–12	50–50–25–10	100
Fiber	11–14	500–300–50–5	100

The potential difference between the charged liquid in the needles and a grounded collector induces fiber jetting in the form of filaments, and upon ejection from the needle into the spinning environment, there is a transition of shearing to elongational flow.^16^ Solid fibers are then formed following solvent evaporation from the filaments.^10^ However, particles are formed at lower solution concentration (electrospraying) whereby lower flow rates and applied voltage, in comparison to fiber formation, were applied in this work (Table2). Here, the liquid drop at the needles' tip becomes charged and the electrostatic repulsion counteracts the surface tension giving a liquid jet,^3^ more specifically at a specific applied voltage and flow rates the drop at the end of the needle tip adopts a conical shape and a fine jet emerges. The jet then breaks up forming small droplets from a “cone-jet,” which is often required to attain fine particle formation.^17^ The continuous break-up of the jet to droplets and accompanying evaporation results in particle formation. Theoretically, it is well accepted that EHD processing in the stable cone-jet mode is very conducive to generating near-monodispersed and spherical particles.

### 2.3 Characterization

The prepared structures were characterized using various techniques including: Scanning electron miscroscopy (SEM): For sectioning fibers, focused ion beam milling (FIB, Carl Zeiss XB1540 “Cross-Beam”) and simultaneous SEM imaging was applied. FIB fibers were collected directly on a glass cover slip and left to air dry prior to analysis. Prior to the FIB studies, dried samples were sputter coated with gold for 60 s. The accelerating voltage ranged from 5 to 10 kV during scanning. Transmission electron microscopy (JEOL-2000FX TEM): Here, following particle collection in medium, a drop was placed on a formvar/carbon on 400 mesh copper TEM grids and then air-dried prior to analysis. TEM studies were performed with an operating voltage of 80 kV and a 10 μm objective aperture to increase mass-thickness contrast. No contrast agent was used and the contrast difference from one layer to another enabled the distinguishing of the different layers. Image J software was used to measure the diameter of about 100 individual particles. All particles in the images were taken into account. A dye release study using UV spectrophoto­metry (UV-2401PC spectrophotometer, Shimadzu) was conducted on the particles. A dye was incorporated in each polymer solution as follows: Layer 1 (L1—innermost layer) contained 40 mg of hematoxylin dye in 10 mL PMSQ solution, layer 2 (L2—second from inside) contained 30 mg Pinacyanol Chloride in PCL solution, layer 3 (L3—third from inside) contained 25 mg pyronin B in PLGA solution and layer four (L4—outermost) contained 25 mg Evans blue in PEG solution of 20:80 water:DCM. Evans blue was miscible in water hence was mixed separately and then added dropwise, under magnetic stirring, to the PEG solution, otherwise it would not dissolve the dye. Upon analysis, the results were normalized to the control at all times. The control sample for normalization contained the four layers of polymer but no dye. The release of dye into simulated body fluid (SBF) was measured at various time points over 28 h. The UV spectra of the dyes, and associated calibration graphs used in the analysis are given in Supporting Information. The characteristic UV wavelengths (nm) used were 320, 348, 280, and 385 for Evans blue, pyronin B, pinacyanol chloride, and hematoxylin, respectively. The reason behind selecting these peaks as references is to have the least influence of each dye on the others' UV measurements.

## 3. Results and Discussion

**Figure 2:**
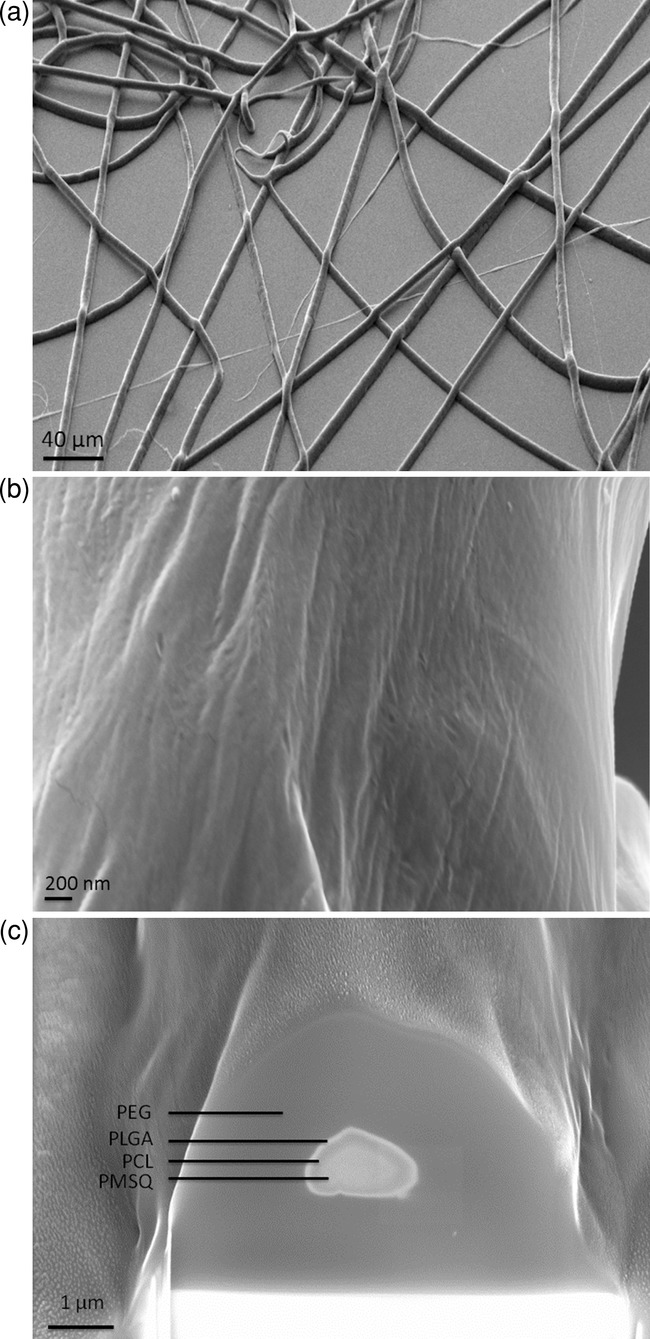
a–c) SEM–FIB microscopic images of four-layered fibers. a) Fibers at low magnification, b) surface morphology of the outer layer, and c) FIB sectioning showing the cross-section of the fibers with four distinct layers, labeled accordingly.

**Figure 3:**
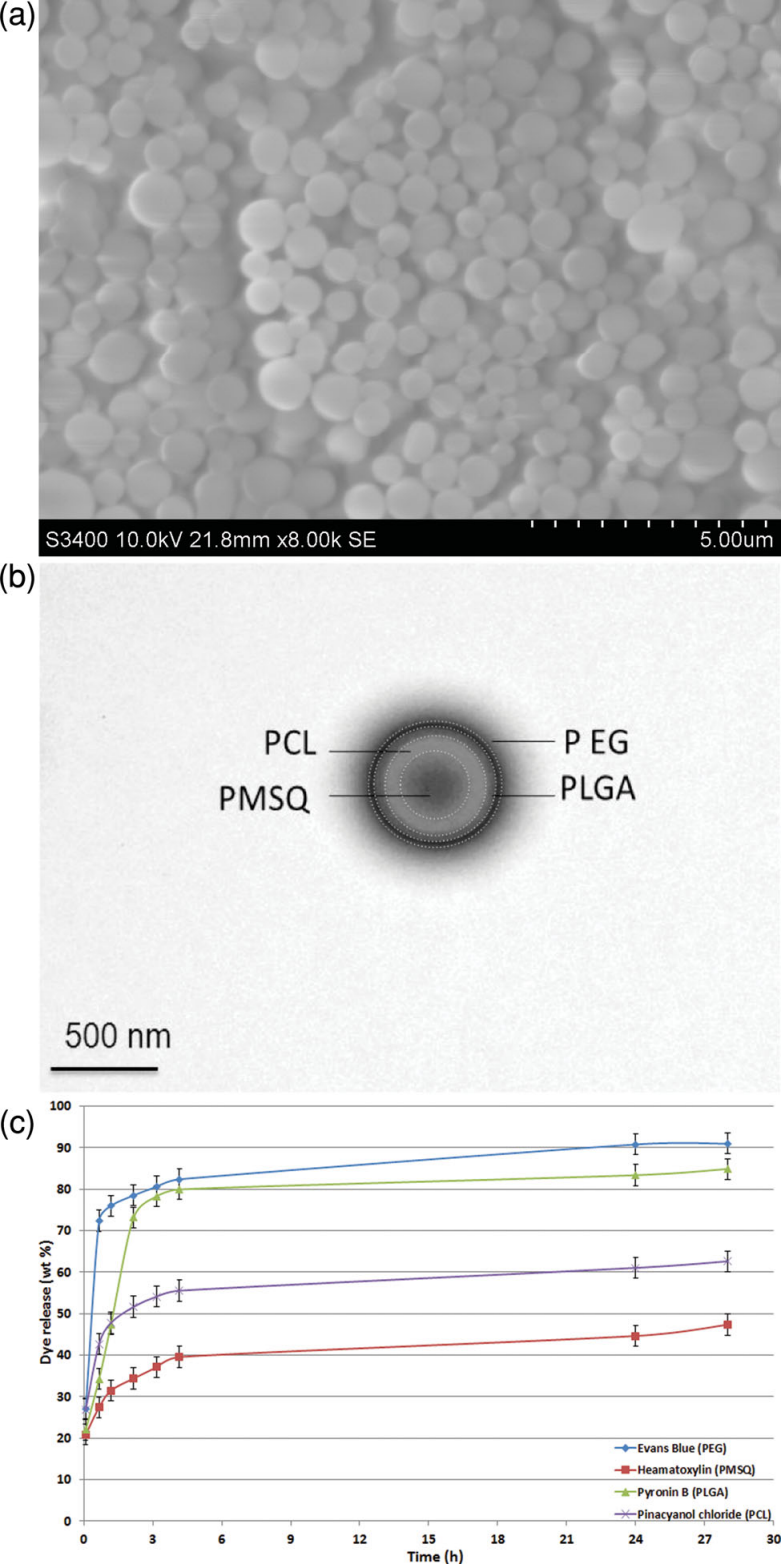
a) SEM image of four-layered particles at low magnification b) Bright-field TEM image of a particle showing four distinct layers. c) Release profile for four layered particle consisting of PEG, PLGA, PCL, and PMSQ loaded with Evans blue, pyronin B, pinacyanol chloride, and hematoxylin, respectively.

In EHD processing, the solution properties such as viscosity, surface tension, and electrical conductivity govern fiber or particle forming. With reference to Table1, for the preparation of both particles and fibers, the surface tension of all four polymer solutions lies within the same range. This facilitates the generation of multilayered structures since the solutions can flow as one and hence interact with one another.^2^ According to Table1, the viscosities of all four solutions vary from one layer to another. It is imperative to combine materials with low viscosity with those that have higher values in order to allow more compact structures and hence achieve more effective encapsulation. In the current study for the forming of both particles and fibers, a high-low-high-low viscosity profile was applied, which was found to be effective in the production of multilayered structures. The current forming route was found to be successful as it resulted in multilayered structures containing the four different polymers: PMSQ–PCL-PLGA– PEG (Figure 2 and 3).

Figure 2a shows, at low magnification, randomly orientated fibers (≈10 μm in diameter) formed with the four needle device. Figure 2b presents the surface morphology and texture of the fibers, showing a relatively rough surface. It must be noted that there was no attempt to design and control the layer thickness by adjusting the needle dimensions or further optimization of the polymer solutions as this was beyond the scope of this report. From Figure 2c, following FIB sectioning, it is clear that the fibers possess four distinct layers, ≈2, 0.07, 0.12, and 1 μm in thickness outer to inner, each layer labeled accordingly. It is well known that the molecular weight of chosen polymers have profound effect on particle/fiber creation. The molecular weight of the polymer indicates the number of entanglement of polymer chains in a solution and hence reflects solution viscosity.^18^ Too low, a molecular weight tends to result in beads and higher molecular weights helps to form fibers with larger diameter.^18^ Low concentrations but high-molecular-weight polymers can maintain sufficient chain entanglements ensuring adequate level of solution viscosity to produce uniform fibers. With increased polymer solution concentration, the fiber dia­meter is thought to increase.^18^–^22^ In addition, the solution flow rates play an important role in fiber diameter such that with decreased flow rate the diameter decreases. It is possible to further optimize the current fibers by altering the solution properties, the choice of polymer and/or solvents to meet specific criteria for various applications. The rough surface observed in this study would suggest a suitable application in tissue engineering scaffolds as this property will promote cell attachment on the surface of the fibers.^19^

Figure 3a presents a SEM image, at low magnification, of the morphology of produced four-layered particles showing smooth, spherical, and nonagglomerated structures. The average size of the particles was found to be 620 ± 150 nm (*n* = 100, where *n* is the number of particles counted) with a polydispersity index of 26%. From the TEM image in Figure 3b, it can clearly be observed that the particles possess a four-layered structure, witnessed by the contrast difference between PEG (L4), PLGA (L3), PCL (L2), and PMSQ (L1), each layer labeled accordingly. It is evident that there is an overall size difference from one layer to another, although like in the case of the fibers, there was no attempt to tailor the device dimensions or the solutions to achieve a specific layer thickness. Nevertheless, it must be noted that this kind of optimization is possible with this method. In the present result, variation between L1 and L4 may be due to the fact that the inner streams break up more effectively than the outer ones under specific voltage and flow rates during processing.^3^ An increase in solution conductivity can also lead to a decrease in diameter due to enhanced coulomb repulsion.^23^

To elucidate the benefits of having four polymeric layers in the particles, a dye release study was conducted. Each layer was loaded with a specific dye and their release was monitored over 28 h (Figure 3c). In presenting release profiles, we take into account dye inevitably lost in the forming process, hence% release is not expected to reach 100%. To compare, loading all the dyes in two types of single polymer particles was also monitored and these dye release curves are given as Supporting Information. These show that other the dye which is specific to that polymer, the release profiles are very much bunched together. Combining four polymers in a particle in this way cannot be done by simple mixing, it is the four-needle EHD coflows that enable the processing and forming of layered particles in this way. Although the selection of polymers and dyes were not predesigned to tailor specific release profiles, it is clear that combining four polymers in this way in particles was very effective in regulating and distributing the dosages of the dyes released as a function of time. Generally, it is expected to show a higher release rate from the outer layers than inner layers. However, this occurrence is very much dependent on the properties of the fabricated particles such as size and layer thickness. Here, overall L4 and L3 were found to be much thinner than L1 and L2 and this showed to have an effect on the initial burst release and also overall release at various time points. It is clear that after 28 h, both Evans blue and pyronin B are released almost completely but only ≈60% and ≈50% of pinacyanol chloride and hematoxylin from the PCL and the PMSQ layer, respectively, are released. This can be attributed to the fact that the PCL layer was thicker and therefore it took longer for the dye to escape and diffuse out into the medium, another benefit of layering.

Dye release is likely to have involved a combination of diffusion and dissolution. The dye is likely to be entrapped within polymeric vesicles that are degradable and hence dissolution would result in the creation of pores that would allow dye to escape. This can be expected for PLGA and PCL, and to a lesser extent in PEG. The polymers would start to degrade as a result of hydrolysis of the polymer chains into biologically safe and progressively smaller moieties. However, this does not apply to PMSQ (L1) and the mechanism can solely be by diffusion through the polymeric membrane. The positive interaction of hydrophilic dye and polymer might limit the tendency of the dye molecules to migrate to the surface of the particles.^19^ The dyes mimic therapeutic agents and it is often required to have a control over the release for maintaining constant levels in the targeted site. This is possible to achieve via the current forming route by altering the solution properties and processing para­meters that determine size, uniformity, and layer thickness of the optimized particles, which forms part of our on-going and future work. A very detailed study on the release capabilities of the products, where the polymers, the geometrical parameters, and the therapeutic agents are systematically varied is also planned. Sufficient physical interactions between the polymer and the dye/drug are essential for getting sustained and prolonged release behavior.^24^,^25^

## 4. Conclusions

In this study, the preliminary experimental findings from a novel four-needle coaxial EHD device are demonstrated. We successfully showed that, for the first time, four-layered structures in both particle and fiber form can be obtained. This is a significant achievement that will boost the biomedical engineering sector striving to generate more efficient therapeutic delivery and novel tissue engineering strategies. The flexibility of EHD forming means that such structures could be optimized for a variety of applications through appropriate selection of solution properties, and/or processing parameters.
