# Supplementary material for: Preparation of Multilayered Polymeric Structures Using a Novel Four-Needle Coaxial Electrohydrodynamic Device
Source: Macromol Rapid Commun. 2014 Feb 8;35(6):618–23. doi: 10.1002/marc.201300777 (PMC4237175; doi:10.1002/marc.201300777)
Supplement: Supplementary file 1 — Supplementary [file marc0035-0618-SD1.pdf]

Copyright WILEY-VCH Verlag GmbH & Co. KGaA, 69469 Weinheim, Germany, 2014.

Supporting Information

for *Macromol. Rapid Commun.*, DOI: 10.1002/marc.201300777

Preparation of Multilayered Polymeric Structures Using a Novel Four-Needle Coaxial Electrohydrodynamic Device

Sheyda Labbaf, Hanif Ghanbar, Eleanor Stride, Mohan Edirisinghe*

Supporting Information

Preparation of multilayered polymeric structures using a novel four-needle coaxial electrohydrodynamic device

Sheyda Labbaf[1], Hanif Ghanbar[1], Eleanor Stride[2,3] and Mohan Edirisinghe[1,2*]

[1]Department of Mechanical Engineering, Biomaterials Research Lab

University College London

Torrington Place, London WC1 7JE (UK)

[2]Department of Mechanical Engineering

University College London

Torrington Place, London WC1E 7JE (UK)

[3]Institute of Biomedical Engineering

Oxford University

Oxford, OX3 7DQ (UK)

***corresponding author: m.edirisinghe@ucl.ac.uk**

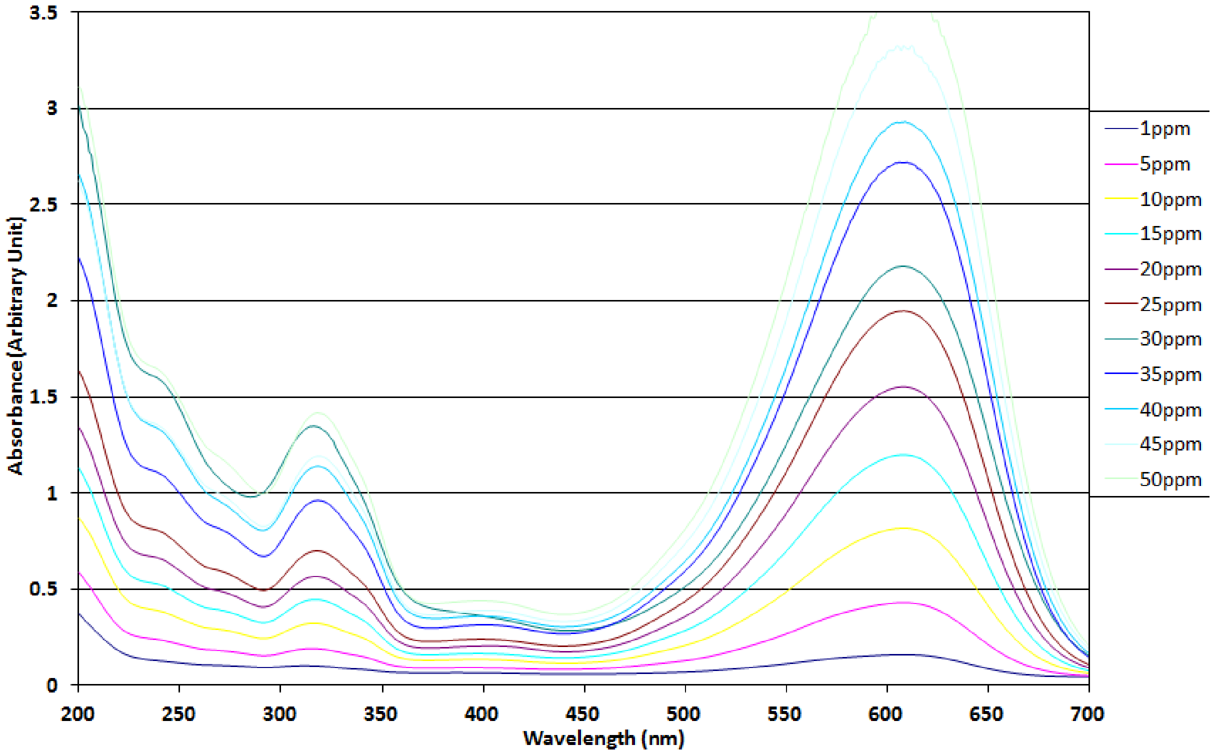

Supporting Information - Evans Blue UV spectra

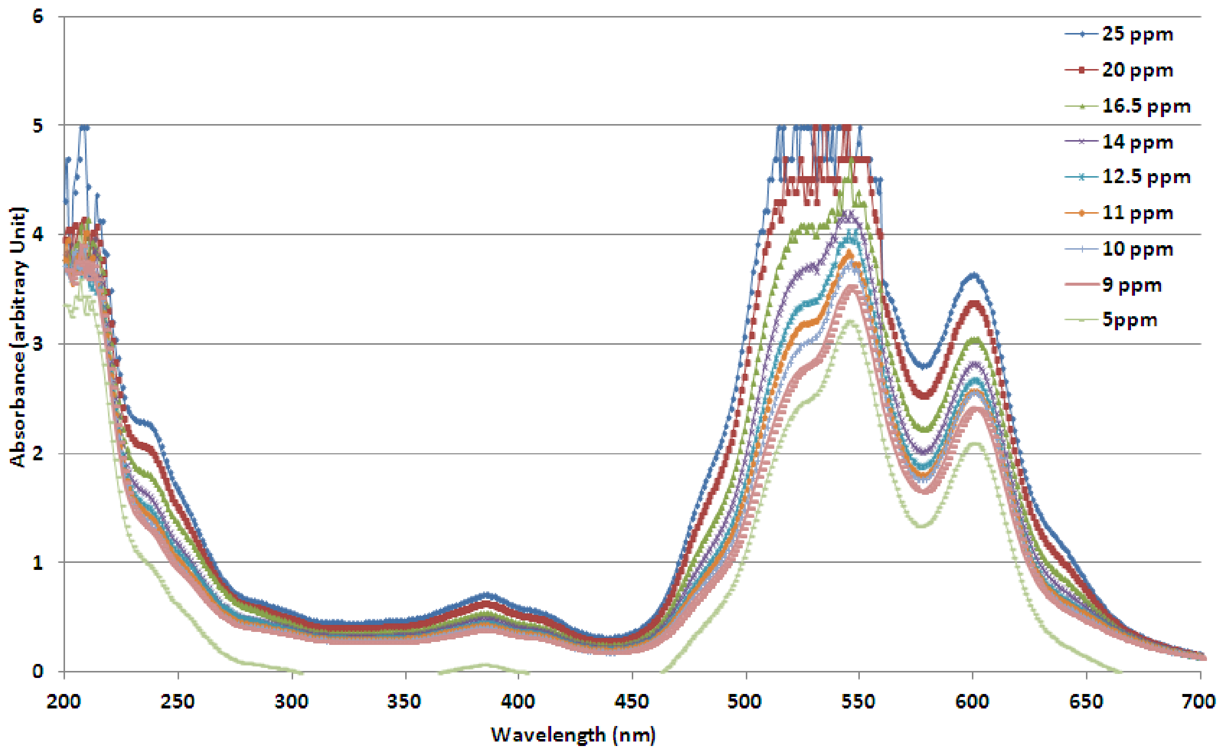

Supporting Information - Hematoxylin UV spectra

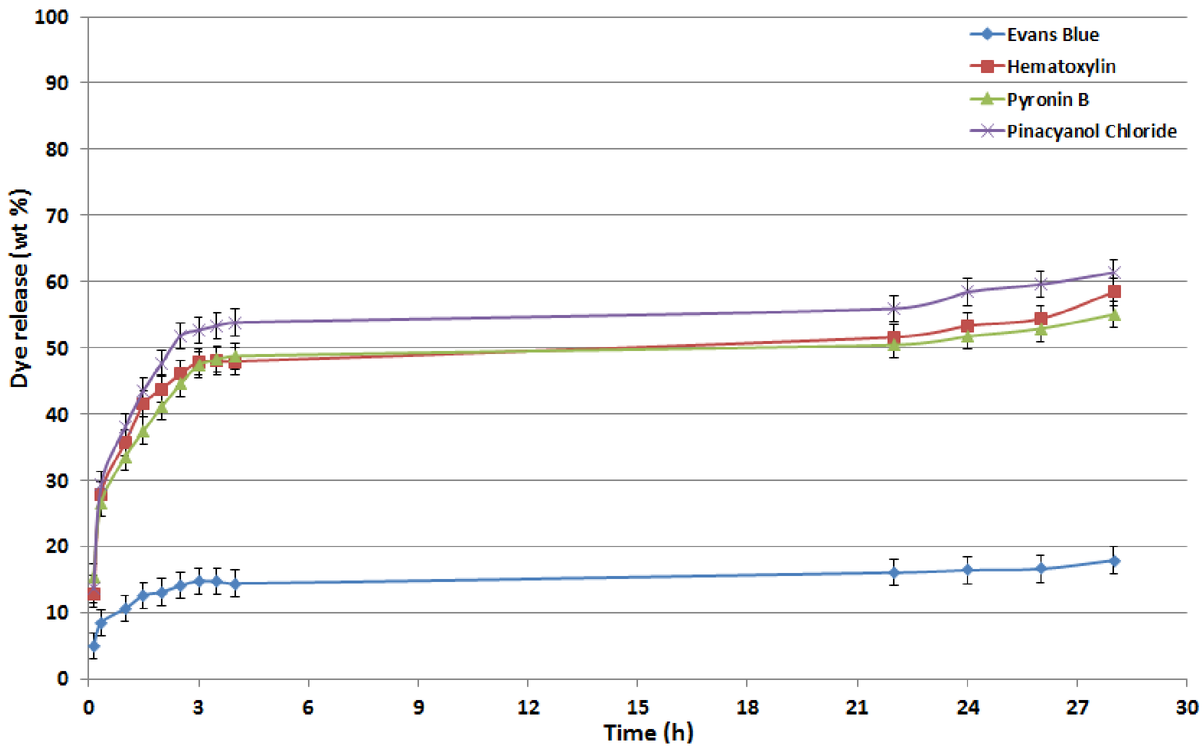

Supporting Information - One polymer (PEG in DCM 10-90) with all four dyes

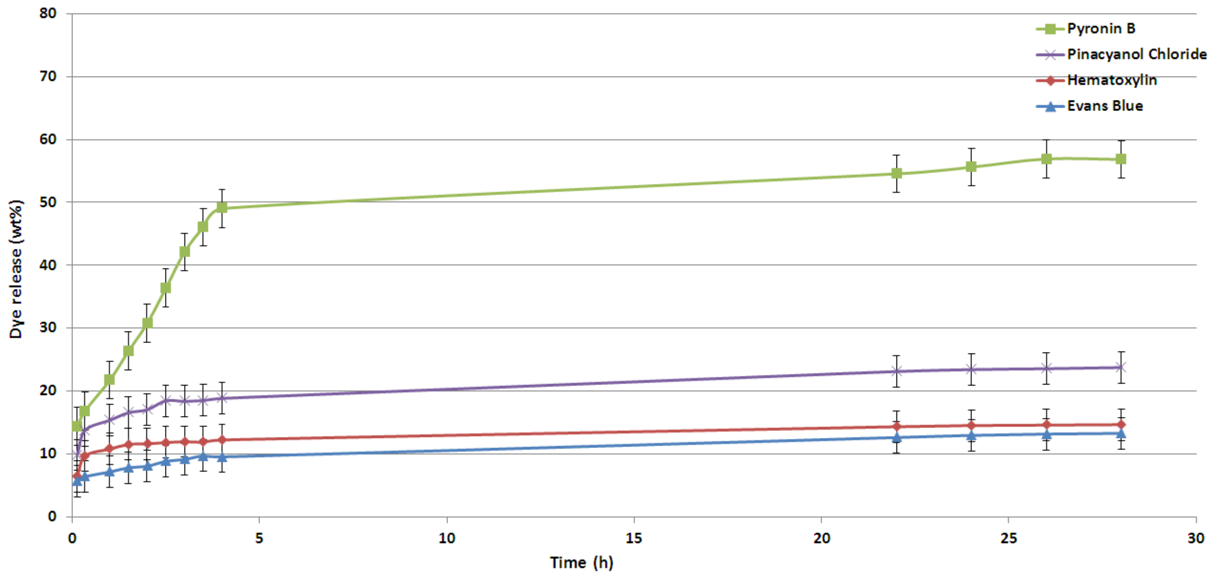

Supporting Information - One polymer (PLGA in DMC 5-95) with all four dyes

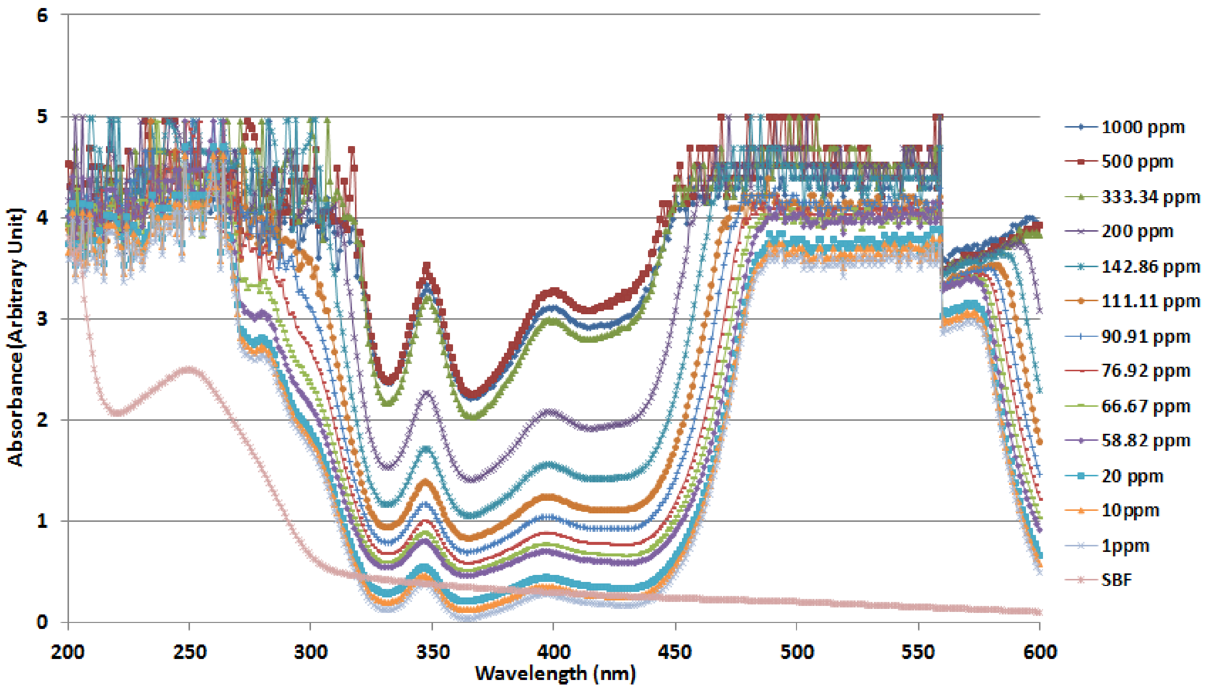

Supporting Information - Pyronin B UV spectra
